# EdgeGuard-AI: Zero-Trust and Load-Aware Federated Scheduling for Secure and Low-Latency IoT Edge Networks

**DOI:** 10.3390/s26061989

**Published:** 2026-03-23

**Authors:** Abdulaziz G. Alanazi, Haifa A. Alanazi

**Affiliations:** Department of Information Systems, Faculty of Computing and Information Technology, Northern Border University, Rafha 91911, Saudi Arabia; abdulaziz.alanazi@nbu.edu.sa

**Keywords:** zero-trust edge computing, trust-aware task scheduling, load-aware offloading, federated learning for edge networks, IoT edge security, adaptive edge resource management

## Abstract

Edge computing is now widely used to support real-time and safety-critical IoT services. However, current edge schedulers usually optimize only performance, while security verification and trust assessment are handled as separate modules. This separation creates a practical risk: tasks may be assigned to lightly loaded but compromised edge nodes, or secure nodes may become overloaded, violating latency requirements. We propose EdgeGuard-AI, a unified trust-driven and load-aware scheduling framework inspired by zero-trust security principles for next-generation IoT edge networks. The framework jointly learns dynamic node trust and short-term workload patterns from distributed edge data and integrates both signals into scheduling decisions. Experimental results on a realistic IoT edge security dataset show a task success rate of 97.3 percent, average scheduling latency of 58.1 ms during stress periods, unsafe offloading below 2 percent, and trust discrimination AUC of 0.971.

## 1. Introduction

The rapid growth of heterogeneous Internet of Things (IoT) infrastructures and mobile edge computing has made distributed learning and task execution a fundamental requirement for modern intelligent services. Recent studies have shown that efficient client scheduling and resource coordination are essential for scalable federated learning in heterogeneous edge environments [1,2,3]. At the same time, large-scale edge systems increasingly support computation-intensive and collaborative services, such as multi-edge content generation and cooperative task processing, which further amplify resource contention and coordination complexity [4,5]. These systems operate under highly dynamic device states, fluctuating workloads, and diverse network conditions, making static or centrally optimized scheduling strategies insufficient for practical deployments.

Security and trust have also become first-order constraints in edge and fog infrastructures. Zero-trust architectures and trust-aware offloading schemes have been proposed to continuously verify device behavior and restrict malicious or unreliable nodes [6,7,8,9]. Federated learning has been adopted to support privacy-preserving and scalable security-aware offloading in sensitive domains such as healthcare and IoMT [10]. In parallel, dynamic resource management and attack-aware allocation strategies have been investigated to defend against persistent and interdependency-based attacks in fog and cloud–edge systems [11,12]. While these studies demonstrate the importance of integrating security mechanisms into edge infrastructures, they mainly focus on either authentication, attack detection, or trust-aware access control, without tightly coupling trust dynamics with real-time load-aware scheduling and distributed learning processes.

Despite these advances, current edge systems still lack a unified and deployable framework that jointly models dynamic trust, workload evolution, and distributed learning constraints when making scheduling decisions. In particular, trust verification is typically applied as a pre-filtering step, and load-aware scheduling is optimized independently, which limits system robustness under adaptive attacks and non-stationary traffic patterns. This paper addresses this gap by proposing EdgeGuard-AI, a unified scheduling framework that jointly models dynamic trust evolution and short-term workload prediction within a single constrained decision loop. Unlike existing approaches that treat trust verification and load-aware scheduling as separate processes, the proposed system integrates both mechanisms directly into the optimization policy, enabling task placement decisions that simultaneously satisfy security and performance constraints.

The main contributions of this work are summarized as follows:

1. We propose a unified zero-trust scheduling framework that jointly integrates adaptive trust modeling and short-term load prediction for secure and low-latency task offloading in heterogeneous IoT edge networks.
2. We design a dynamic trust inference mechanism that continuously updates node reliability from traffic evidence and constrains scheduling decisions under explicit trust admissibility conditions.
3. We develop a load-aware scheduling policy that directly couples predicted workload states with trust scores, enabling robust task placement under bursty traffic and adversarial behaviors.
4. We introduce a federated training architecture that supports privacy-preserving learning of security and performance models across distributed and heterogeneous edge sites.
5. We conduct extensive experimental evaluation under attack scenarios, client heterogeneity, and workload shifts, demonstrating consistent improvements in task success rate, latency, and unsafe offloading reduction compared with strong baselines.

The paper is structured as follows: Section 2 discusses related work, followed by Section 3, which presents the problem statement to justify the necessity of the work. Section 4 describes the dataset and preprocessing steps used in this research. Section 5 presents the proposed method, EdgeGuard-AI. Section 6 reports the results obtained from this work, and Section 7 concludes the paper and outlines future research directions.

## 2. Related Work

Recent research has increasingly emphasized the role of zero-trust principles and federated learning for improving the security of edge computing systems. Almuseelem [10] proposed a secure and latency-aware task offloading framework for Internet of Medical Things environments by combining federated learning with a zero-trust access model. Their work demonstrated that authentication and trust enforcement can be integrated with offloading decisions to reduce response delay. Similarly, Ali et al. [13] introduced a maturity framework that formalizes how zero-trust security concepts can be gradually adopted in multi-access edge computing infrastructures. Sedjelmaci and Ansari [6] further extended this direction by embedding attack detection modules inside a zero-trust architecture for 6G edge networks, showing that continuous verification significantly improves resilience against advanced attacks.

In parallel, several studies have focused on improving performance and reliability of task processing at the edge through intelligent resource and offloading control. Zou et al. [14] investigated the computation–transmission tradeoff for minimizing information staleness using queue-aware optimization. Deng et al. [15] explored software-orchestrated and hardware-accelerated artificial intelligence pipelines to reduce inference latency in edge systems. Reinforcement learning has also been widely applied to adaptive offloading and system coordination, such as in digital-twin-assisted offloading optimization [16] and federated ensemble reinforcement learning for distributed edge environments [17]. These approaches significantly improve latency and throughput, but they primarily optimize performance metrics without explicitly embedding security or trust constraints into the scheduling policy.

Another important line of work addresses attack detection and load balancing for IoT and edge platforms. Kumar and Singh [18] proposed a reinforcement learning framework to detect and mitigate DDoS attacks in IoT edge systems. Devi et al. [19] introduced a deep learning-based load balancing mechanism with enhanced security for cloud–IoT environments. However, these methods treat security monitoring and load management as two loosely coupled components, and the scheduling decisions are not directly constrained by a continuously updated trust model. As a result, performance optimization and security enforcement remain largely independent in the decision loop.

Trust-aware offloading has also received increasing attention. Kong et al. [20] proposed a multifeedback trust mechanism to improve reliability of task offloading in IoT edge computing, while Liu et al. [21] developed a reinforcement learning-based trust-aware offloading strategy for UAV-assisted edge systems. These studies show that trust information can improve task reliability and cost efficiency. Nevertheless, the trust models are typically designed as static or slowly updated indicators and are not tightly integrated with adaptive load prediction, federated learning, or zero-trust verification pipelines.

Despite these advances, an important gap remains. Existing zero-trust frameworks mainly focus on access control and attack detection [6,13], while intelligent scheduling and offloading methods focus on latency, freshness, or throughput [14,16,17]. Trust-aware offloading studies [20,21] do not explicitly address federated deployment constraints and rapid trust dynamics under evolving attacks. Moreover, current approaches rarely unify zero-trust verification, adaptive trust modeling, and load-aware scheduling within a single optimization framework. The proposed EdgeGuard-AI directly addresses this gap by jointly learning dynamic trust scores and short-term workload patterns, and by embedding these signals into a zero-trust constrained scheduling policy trained under a federated setting. This unified design enables secure and low-latency task execution while preserving scalability and privacy across heterogeneous edge sites.

While existing studies address important aspects of secure edge computing, most approaches focus on a single dimension of the problem. Trust-aware offloading methods typically rely on static or slowly updated trust indicators and do not incorporate real-time workload dynamics when making scheduling decisions. Conversely, performance-oriented scheduling frameworks optimize latency, resource utilization, or throughput but often assume trusted infrastructure and therefore lack continuous security verification mechanisms.

The proposed EdgeGuard-AI framework differs from these approaches by jointly modeling two key system dynamics: (i) the evolution of node trust derived from observed traffic evidence and (ii) the short-term workload conditions of edge nodes. These signals are integrated within a constrained optimization loop that enforces trust admissibility and risk constraints during task assignment. In addition, the framework supports federated learning across heterogeneous edge sites, allowing the scheduling policy to adapt across distributed environments without sharing raw traffic data.

Recent survey studies have also examined the security challenges associated with machine learning and distributed intelligence in critical infrastructure systems such as smart grids [22,23]. For example, recent reviews have analyzed vulnerabilities of machine learning techniques deployed in IoT-enabled smart-grid environments and highlighted the risks associated with adversarial manipulation, unreliable edge devices, and distributed control architectures. Other surveys have investigated cyber-resiliency strategies for distributed energy resource (DER)-based smart grids, emphasizing the importance of trustworthy and resilient computation across heterogeneous and geographically distributed nodes. These studies reinforce the need for secure and adaptive decision mechanisms in edge-based infrastructures, further motivating trust-aware and resilient scheduling frameworks such as the one proposed in this work.

Table 1 highlights that the proposed framework uniquely combines dynamic trust modeling, load-aware scheduling, and federated deployment within a unified optimization-based scheduling policy.

## 3. Problem Statement

Modern IoT edge networks execute delay-sensitive and safety-critical tasks under highly dynamic traffic, heterogeneous devices, and continuous cyber threats. In practice, most existing edge schedulers treat security verification and load-aware task scheduling as two independent processes. This separation causes a fundamental deployment gap: a task can be scheduled to a lightly loaded edge node that is already compromised or unreliable, or a secure node can become overloaded and violate latency constraints. Our experimental observations in Section 6 confirm that this decoupled design leads to high unsafe offloading rates and unstable latency during attack bursts and workload surges.

Let $E=\{e_{1},\ldots ,e_{N}\}$ denote the set of edge nodes and let $T_{t}$ be the set of tasks arriving at time slot *t*. For each task $\tau \in T_{t}$ and node $e_{j}$, the scheduler must jointly consider the predicted workload state and the trustworthiness of the node. This joint decision problem can be expressed as

(1) $$e^{*}\left(\tau \right)=\arg\min_{e_{j}\in E}\;E\;\left[L(\tau ,e_{j},t)\right]\;s.t.\;S\left(e_{j},t\right)\geq \delta ,$$

where $L(\tau ,e_{j},t)$ denotes the expected end-to-end latency of assigning task τ to node $e_{j}$ at time *t*, $S(e_{j},t)$ is the security trust score of node $e_{j}$, and δ is the minimum acceptable trust threshold. Equation (1) shows that a feasible scheduling decision must satisfy both performance and security constraints at the same time.

However, in real deployments, both $L(\tau ,e_{j},t)$ and $S(e_{j},t)$ are unknown and change rapidly due to workload bursts, network dynamics, and adaptive attacks. Moreover, edge data are naturally distributed across sites and cannot be centrally collected due to privacy and bandwidth constraints. This makes centralized learning unreliable and exposes the system to client and domain shifts, as reflected by the federated instability observed in baseline methods in Section 6.

Therefore, the core problem addressed in this paper is to design a unified and deployable framework that can (i) continuously learn trustworthy behavior of edge nodes, (ii) accurately predict short-term load dynamics, and (iii) schedule tasks under a strict zero-trust constraint in a distributed manner. This motivates the proposed EdgeGuard-AI framework, which tightly integrates adaptive trust modeling, load-aware prediction, and federated learning to produce secure and low-latency scheduling decisions, as validated by the robustness, convergence, and attack-time performance reported later in this paper.

## 4. Dataset and Preprocessing

This study is conducted using the Edge-IIoTset dataset, a large-scale, publicly available benchmark specifically designed for security research in IoT and edge computing environments. The dataset was generated from a realistic multi-layer testbed that integrates IoT sensors, industrial controllers, edge gateways, and cloud services. Traffic is captured across heterogeneous protocols, including TCP/IP, UDP, HTTP, MQTT, Modbus, and DNS, reflecting the communication diversity found in real deployments. Edge-IIoTset includes both benign operational traffic and a wide spectrum of cyber-attacks, such as DoS/DDoS, reconnaissance, injection, spoofing, man-in-the-middle, and malware-driven behaviors. These properties make it suitable for studying adaptive zero-trust security and load-aware decision systems, where models must respond to both malicious activity and workload fluctuations.

Formally, the dataset is defined as

(2) $$D={\left\{\left(x_{i},y_{i},c_{i},t_{i}\right)\right\}}_{i=1}^{N},$$

where $x_{i}\in R^{F}$ is the feature vector extracted from the *i*-th network flow, $y_{i}$ is the corresponding security label, $c_{i}$ denotes the edge client or device group, and $t_{i}$ represents the capture timestamp. This representation preserves not only traffic statistics, but also client identity and temporal structure, which are essential for modeling adaptive scheduling and federated learning behavior. Each feature vector aggregates packet-level information into flow-level descriptors such as packet counts, byte volumes, inter-arrival times, protocol flags, and resource utilization indicators.

The original Edge-IIoTset release contains more than one thousand raw attributes per flow. Following the dataset authors’ recommendations and prior empirical validation, we retain a subset of 61 high-correlation features that capture transport behavior, protocol semantics, and temporal dynamics while avoiding redundancy. Table 2 summarizes the dataset scale and composition before preprocessing.

All packet traces are first converted into bidirectional network flows using a fixed temporal window ΔT. Packets exchanged between the same source–destination pair within ΔT are merged into a single flow instance. This step aligns the data with real edge monitoring systems, where decisions are typically made at the flow level rather than on individual packets. Flows containing incomplete headers, undefined protocol fields, or corrupted values are removed. Missing numerical attributes are imputed using median statistics computed on the training split to avoid bias introduced by extreme values.

To stabilize training and prevent dominance of high-magnitude features, all numeric attributes are standardized using z-score normalization:

(3) $${\hat{x}}_{i}=\frac{x_{i}-\mu }{\sigma },$$

where μ and σ are the per-feature mean and standard deviation estimated from the training data. This transformation enforces comparable feature scales, accelerates convergence, and ensures that trust estimation and scheduling policies are not driven by measurement units.

Edge-IIoTset exhibits strong class imbalance, with benign traffic significantly outweighing several attack categories. To mitigate biased learning, we apply a hybrid balancing strategy that combines minority oversampling and controlled majority undersampling. Let $D_{b}$ and $D_{m}$ denote benign and malicious subsets. The resampled training set is constructed such that

(4) $$|D_{b}\left|\;\approx \;\right|D_{m}|,$$

while preserving the internal proportions of individual attack types. This process ensures that the model learns discriminative patterns for both rare and frequent threats. In addition to categorical labels, we derive a continuous risk intensity signal from traffic rate, anomaly concentration, and resource stress indicators, which later guides adaptive scheduling.

To support zero-trust modeling and federated learning evaluation, samples are grouped into client partitions ${\left\{D_{k}\right\}}_{k=1}^{K}$ based on device type, subnet origin, and traffic profile. Each client partition simulates an independent edge node with non-identical data distributions, reflecting the heterogeneous traffic patterns and attack prevalence typically observed in real-world edge deployments. Temporal ordering is preserved so that workload bursts and attack campaigns remain coherent. This allows the proposed system to learn policies that adapt both to trust variation and to dynamic load conditions.

After preprocessing, the refined dataset is represented as

(5) $$\hat{D}={\left\{\left({\hat{x}}_{i},y_{i},c_{i},t_{i},r_{i}\right)\right\}}_{i=1}^{N^{\prime }}$$

where $r_{i}$ denotes the derived continuous risk indicator. The dataset is then split into training (70%), validation (10%), and testing (20%) sets using stratified sampling across both labels and clients. Table 3 summarizes the dataset after preprocessing.

This multi-stage preprocessing pipeline converts raw packet captures into structured, balanced, and client-aware learning inputs. It enables EdgeGuard-AI to jointly reason about malicious behavior, trust variation, and workload dynamics, providing a realistic foundation for evaluating adaptive zero-trust security and load-aware scheduling strategies.

## 5. Proposed Method: EdgeGuard-AI

This section presents EdgeGuard-AI, an adaptive framework that couples zero-trust security with load-aware scheduling for IoT edge networks. The key idea is simple: every scheduling decision must be trust-feasible and load-feasible at the same time. In real edge deployments, traffic patterns and attacks change quickly, and edge nodes often have limited compute and memory. Therefore, static access control alone is not enough, and pure performance-based scheduling is unsafe. EdgeGuard-AI solves this by (i) continuously computing trust from flow evidence, (ii) forecasting near-term load, and (iii) selecting task placements under explicit trust and risk constraints. The full pipeline is implemented using four connected algorithms (Algorithms 1–4), where the output of each stage is the input to the next stage.

We use the preprocessed dataset $\hat{D}$ defined in Equation (5). Each sample contains a normalized flow feature vector ${\hat{x}}_{i}$, a security label $y_{i}$, a client identifier $c_{i}$, a timestamp $t_{i}$, and a continuous risk indicator $r_{i}$. The goal is not only to classify attacks, but to convert traffic evidence into a *trust signal* and use it for safe scheduling. This requires the model to preserve time order and client identity because trust and load are both time-varying.

Algorithm 1 defines how EdgeGuard-AI computes trust from streaming traffic. For each flow sample, an evidence vector $z_{i}$ is built from the normalized flow features and the derived risk indicator. In practice, $z_{i}$ contains compact, decision-friendly signals such as traffic burstiness, protocol behavior, anomaly concentration, and resource stress indicators. A suspicion score $s_{i}$ is computed using a logistic mapping,

(6) $$s_{i}=\sigma \left(\omega^{⊤}z_{i}\right),$$

where σ(·) is the sigmoid function and ω is a learnable weight vector. Equation (6) ensures $s_{i}\in \left(0,1\right)$, which makes the value suitable for trust updates and constraint checks.
**Algorithm 1:** EdgeGuard-AI (Part I): Zero-Trust Evidence Construction and Trust Scoring
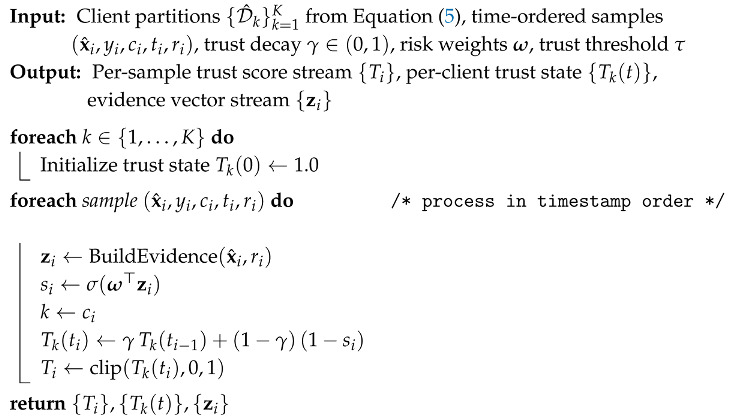


**Algorithm 2:** EdgeGuard-AI (Part II): Load Modeling and Scheduling-State Formation

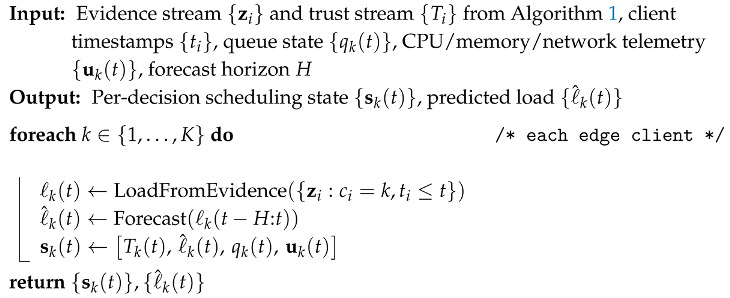



**Algorithm 3:** EdgeGuard-AI (Part III): Trust-Constrained Load-Aware Scheduling Policy

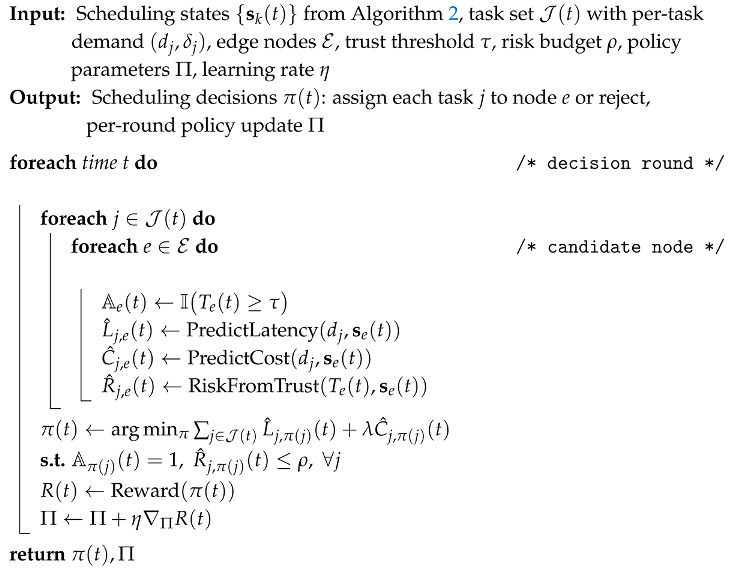



### 5.1. Evidence Vector Construction

The evidence vector $z_{i}$ summarizes the security- and workload-relevant signals extracted from the normalized flow representation ${\hat{x}}_{i}$ and the derived risk indicator $r_{i}$. Instead of using the entire high-dimensional feature space directly, EdgeGuard-AI constructs a compact evidence representation composed of aggregated traffic statistics and behavioral indicators. In particular, the evidence vector includes: (i) traffic burst metrics such as packet rate and inter-arrival variance, (ii) protocol behavior indicators derived from TCP/UDP flags and session statistics, (iii) anomaly concentration features capturing deviations from typical flow distributions, and (iv) resource stress indicators reflecting CPU, memory, or queue pressure associated with the observed flows.
**Algorithm 4:** EdgeGuard-AI (Part IV): Federated Adaptation with Secure Aggregation
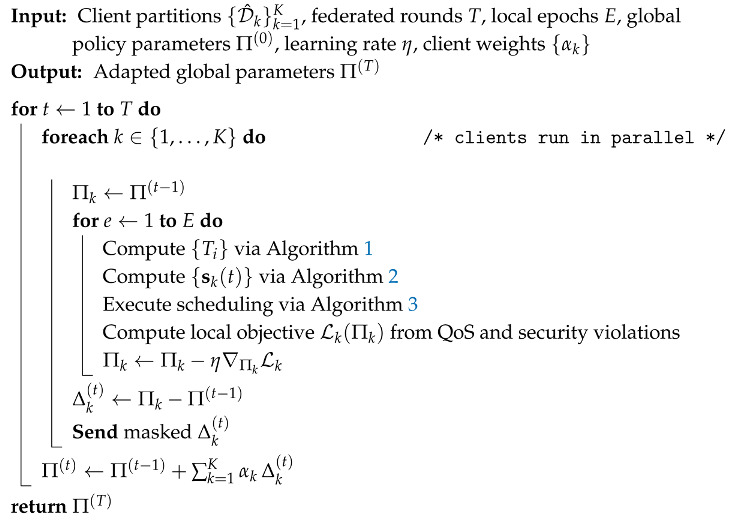


Empirically, the most influential components for trust estimation correspond to anomaly concentration and burst-level traffic statistics, as these features are strongly correlated with malicious activity such as scanning, flooding, or injection attacks. By combining these signals with the derived risk indicator $r_{i}$, the evidence vector provides a compact representation that allows the suspicion score in Equation (6) to capture both instantaneous anomalies and sustained behavioral deviations.

The per-client trust state is then updated using exponential forgetting:

(7) $$T_{k}\left(t_{i}\right)=\gamma \;T_{k}\left(t_{i-1}\right)+\left(1-\gamma \right)\;\left(1-s_{i}\right),$$

where $k=c_{i}$ and γ∈(0,1) is the decay factor. Equation (7) is important for two reasons. First, it supports *continuous verification*: trust changes as new evidence arrives. Second, it avoids overreacting to a single noisy flow because the trust state is smoothed over time. The emitted trust score $T_{i}$ is clipped to [0,1] to maintain a consistent decision range. This trust score becomes the security gate used by the scheduling policy in Part III.

Edge scheduling decisions must consider both current resource usage and near-future load. Algorithm 2 builds this capability. For each client *k*, EdgeGuard-AI forms a workload sequence $\ell_{k}\left(t\right)$ from recent evidence. Intuitively, $\ell_{k}\left(t\right)$ captures how much traffic or task demand is arriving at the edge node at time *t*. Because edge traffic is bursty, we forecast the short-term load:

(8) $${\hat{\ell }}_{k}\left(t\right)=\mathrm{Forecast}\left(\ell_{k}\left(t-H:t\right)\right),$$

where *H* is the look-back horizon. Equation (8) reduces instability in scheduling decisions by preventing aggressive oscillation when traffic spikes occur.

The scheduler state is then built by composing trust, predicted load, queue occupancy, and device telemetry:

(9) $$s_{k}\left(t\right)=\left[T_{k}\left(t\right),\;{\hat{\ell }}_{k}\left(t\right),\;q_{k}\left(t\right),\;u_{k}\left(t\right)\right].$$

Equation (9) is the key interface between security and performance. It ensures that every edge node is represented by a compact state that includes both *security readiness* (trust) and *service capacity* (load and resources). The state $s_{k}\left(t\right)$ becomes the input to the constrained scheduling policy.

Algorithm 3 defines the scheduling policy. At each decision time *t*, a set of tasks J(t) arrives, where each task *j* has a demand profile $d_{j}$ and a deadline or delay tolerance $\delta_{j}$. EdgeGuard-AI evaluates each candidate node *e* and checks whether it is admissible under zero-trust:

(10) $$A_{e}\left(t\right)=I\left(T_{e}\left(t\right)\geq \tau \right),$$

where τ is a trust threshold and I(·) is the indicator function. Equation (10) is a strict security gate: nodes below τ are not allowed to receive sensitive tasks, even if they have spare capacity.

For admissible nodes, the scheduler predicts latency and cost, and computes a risk score driven by trust:

(11) $${\hat{R}}_{j,e}\left(t\right)=\mathrm{RiskFromTrust}\left(T_{e}\left(t\right),s_{e}\left(t\right)\right).$$

Equation (11) is used to enforce a risk budget. The final decision selects task-to-node assignments by optimizing QoS while satisfying security constraints:

(12) $$\begin{array}{cc} \pi (t) & =\arg\min_{\pi }\sum_{j\in J(t)}\left({\hat{L}}_{j,\pi (j)}\left(t\right)+\lambda \;{\hat{C}}_{j,\pi (j)}\left(t\right)\right),\\ s.t.\; & A_{\pi (j)}\left(t\right)=1,\;{\hat{R}}_{j,\pi (j)}\left(t\right)\leq \rho ,\;\forall j\in J\left(t\right). \end{array}$$

Equation (12) forms the core decision rule of the proposed framework, jointly optimizing latency and cost while enforcing trust and risk constraints: trust and risk are constraints, while latency and cost are optimized objectives. This structure is practical because it allows safe operation even under load spikes. If no node satisfies the constraints, the task can be delayed, downgraded, or rejected, which prevents unsafe offloading.

Edge environments are distributed, and data sharing is often restricted. Algorithm 4 provides a federated adaptation mechanism so that EdgeGuard-AI can learn across clients without sharing raw traffic records. Each client *k* starts from the global policy parameters $\Pi^{\left(t-1\right)}$, runs local rollouts by executing Algorithms 1–3, and updates its local parameters using a combined objective that penalizes QoS loss and security violations.

The client then sends an update $\Delta_{k}^{\left(t\right)}$ to the server, and the server aggregates using a FedAvg-style rule:

(13) $$\Pi^{\left(t\right)}=\Pi^{\left(t-1\right)}+\sum_{k=1}^{K}\alpha_{k}\;\Delta_{k}^{\left(t\right)},$$

where $\alpha_{k}$ are client weights, typically proportional to local data size. Equation (13) is important for practical deployment because it supports continuous improvement under client shift. Edge nodes may differ in device types, traffic profiles, and attack exposure, so federated adaptation helps the global policy remain robust across heterogeneous conditions.

After preprocessing the Edge-IIoTset dataset, we obtain a clean and structured flow-level dataset $\hat{D}$ where each record is a normalized feature vector with its label, client identity, timestamp, and a derived risk indicator. In simple terms, the raw packets are converted into fixed-size flow vectors, noisy and broken rows are removed, features are standardized so that one feature does not dominate another, and imbalance is reduced so that rare attacks are not ignored. We also keep client partitions and time order because our method needs to learn how trust and load change over time at each edge node. This final dataset becomes the direct input stream for Algorithm 1. In Algorithm 1, each flow sample is transformed into a compact evidence vector $z_{i}$ that summarizes the security-relevant and load-relevant signals. From $z_{i}$, the model computes an instantaneous suspicion value $s_{i}$ using Equation (6), and then updates the client trust state using Equation (7). The key point is that trust is not a fixed label; it is continuously updated as new flows arrive. Therefore, the output of Algorithm 1 is a time-varying trust score $T_{k}\left(t\right)$ per client and a trust value $T_{i}$ per sample, which represents the current security confidence of that edge node under observed traffic.

Next, Algorithm 2 takes the trust stream from Algorithm 1 and combines it with the system load signals. It builds a workload sequence from recent evidence, predicts near-future load using Equation (8), and then forms the final scheduling state vector $s_{k}\left(t\right)$ using Equation (9). This state is a single compact input that contains trust, predicted load, queue occupancy, and resource telemetry, so it fully describes whether an edge node is both safe and capable at the current time. Algorithm 3 then uses these states to schedule tasks. For each node, it first checks admissibility using Equation (10), which enforces zero-trust by blocking nodes below the trust threshold. For the remaining nodes, it predicts latency and resource cost, computes a risk score using Equation (11), and then selects the task placement policy π(t) by solving the constrained objective in Equation (12). This step is the core of our contribution because it ties security and scheduling into one decision rule: tasks are placed only when trust and risk constraints are satisfied, and among safe choices the policy prefers lower latency and lower cost. Finally, Algorithm 4 makes the entire system adaptive across multiple edge sites. Each client trains locally using the same trust, load, and scheduling loop, then sends only parameter updates to a server. The server aggregates them using Equation (13). This allows EdgeGuard-AI to improve over time under client shift, without sharing raw traffic data, while keeping the same zero-trust and load-aware behavior.

**Figure 1:**
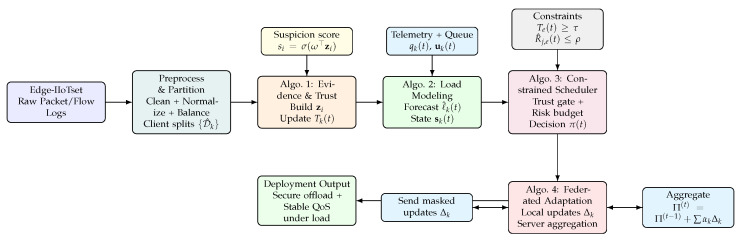
EdgeGuard-AI overview: the preprocessed Edge-IIoTset streams are converted into zero-trust evidence and time-varying trust (Algorithm 1), fused with load forecasting into a scheduling state (Algorithm 2), and used for trust- and risk-constrained task placement (Algorithm 3). Federated adaptation (Algorithm 4) updates the global policy without sharing raw traffic.

Figure 1 summarizes the complete EdgeGuard-AI pipeline and highlights how the dataset stream is transformed into trust signals, load-aware states, constrained scheduling actions, and federated policy updates. The trust scoring, state formation, and constrained decision steps correspond to Algorithms 1–3, while the privacy-preserving adaptation loop is defined in Algorithm 4.

### 5.2. Implementation Details and Parameter Settings

To improve reproducibility, we summarize the main implementation parameters used in the EdgeGuard-AI framework. The trust decay factor in Equation (7) is set to γ=0.9, which provides a balance between stability and responsiveness to new evidence. The short-term load forecasting horizon in Equation (8) is set to H=10 recent time steps.

For scheduling decisions, the admissibility threshold in Equation (10) is set to τ=0.7, which was selected based on validation experiments balancing security filtering and system throughput. The risk budget parameter in Equation (12) is set to ρ=0.3, which limits the probability of assigning tasks to nodes with elevated security risk.

Federated training follows a synchronous aggregation process with T=30 communication rounds and E=5 local epochs per client. The learning rate for policy updates is set to $\eta =10^{-3}$. Client aggregation weights $\alpha_{k}$ are proportional to the number of local samples in each client partition.

All experiments were implemented in Python 3.10 using standard scientific computing libraries, and scheduling decisions were executed sequentially over timestamp-ordered traffic flows to emulate an online edge deployment environment.

### 5.3. Threat Model Scope

The zero-trust principle adopted in EdgeGuard-AI focuses on continuous verification of edge nodes based on observed traffic behavior and dynamically updated trust scores. Nodes are therefore not assumed to be trustworthy by default and must satisfy the admissibility constraint defined in Equation (10) before receiving tasks.

The current framework primarily addresses compromised or unreliable edge nodes whose behavior can be inferred from network evidence. We do not explicitly model adversarial federated clients performing model poisoning, gradient manipulation, or collusion during the federated aggregation process. Handling Byzantine or adversarial client behavior in federated learning would require additional robust aggregation mechanisms and is considered an important direction for future work.

## 6. Results

### 6.1. Experimental Environment

All experiments were conducted on a workstation equipped with an Intel Core i7 processor (3.6 GHz), 32 GB RAM, and an NVIDIA RTX-series GPU used for model training and evaluation. The proposed framework was implemented in Python using common machine learning and data processing libraries including PyTorch 2.9, NumPy 2.2, and Scikit-learn 1.7, and executed under a Linux-based environment.

To evaluate scheduling behavior under realistic traffic conditions, we used a trace-driven simulation based on the Edge-IIoTset dataset described in Section 4. The dataset was replayed in chronological order to preserve temporal workload dynamics and attack bursts. Edge nodes were simulated as independent clients with heterogeneous traffic distributions, enabling evaluation of the federated adaptation mechanism under non-IID client settings.

The computational overhead of EdgeGuard-AI arises primarily from trust estimation, short-term workload forecasting, and constrained scheduling decisions. These operations rely mainly on lightweight vector computations and short sliding-window models. In practice, the scheduling decision is computed within a few milliseconds per decision round, making the framework suitable for latency-sensitive edge environments.

### 6.2. Evaluation Protocol

To ensure statistical reliability, all experiments were repeated multiple times using different randomized task arrival orders and client sampling during federated training. The reported values correspond to the mean performance across runs, and variability is reported using the standard deviation. This evaluation protocol helps verify that the observed improvements of EdgeGuard-AI are consistent and not due to a single execution instance.

This section evaluates the effectiveness of EdgeGuard-AI in detecting malicious traffic patterns and generating reliable trust signals for secure task scheduling. The experiments focus on both detection accuracy and the stability of the resulting trust scores, which directly influence scheduling decisions. All results are reported on the test split of the Edge-IIoTset dataset using the client-aware and time-preserving partition described in Section 4. Although EdgeGuard-AI is not designed as a standalone intrusion detector, the quality of its evidence representation directly affects trust estimation and scheduling decisions. We therefore first evaluate the classification performance produced by the evidence and security head used inside Algorithm 1.

Although the evaluation uses traffic traces from the Edge-IIoTset dataset, the scheduling policy operates in a sequential closed-loop manner that emulates online edge deployment. Specifically, the test data are processed in strict timestamp order, and at each decision step the scheduler receives newly observed flow evidence, updates the trust state using Equation (7), predicts the short-term workload using Equation (8), and then performs task assignment according to the constrained optimization in Equation (12). The resulting scheduling decision modifies the system state for the next time step, including queue occupancy and node workload conditions. This sequential execution ensures that scheduling decisions depend only on information available at that moment in time, closely matching the behavior of a real-time edge scheduler operating on live traffic streams. Trace-driven evaluation is used primarily to provide reproducibility and controlled attack injection while preserving realistic temporal dynamics.

Table 4 shows that the proposed evidence head achieves the highest overall performance. The improvement is mainly due to the joint use of flow statistics, temporal dynamics, and risk indicators within the evidence vector $z_{i}$ constructed in Algorithm 1. This confirms that the extracted evidence is sufficiently discriminative to support reliable downstream trust modeling.

The main objective of EdgeGuard-AI is not only to detect attacks, but to generate a continuous trust score that reflects the real security condition of each edge client over time. The trust signal is computed using the exponential update in Equation (7). We evaluate the trust score by measuring its ability to separate benign and malicious traffic and its temporal stability.

Table 5 shows that the trust score strongly separates benign and malicious flows $T_{i}$ strongly separates benign and malicious flows. The false-trust rate measures how often malicious samples are incorrectly assigned a trust value above the admissibility threshold τ in Equation (10). A low value indicates that unsafe nodes are rarely considered eligible for task execution. The trust detection delay reports the average time required for the trust state $T_{k}\left(t\right)$ to drop below τ after an attack begins. This confirms that the exponential update in Equation (7) reacts rapidly while still avoiding unstable oscillations, as reflected by the low variance under benign conditions.

Since EdgeGuard-AI is designed for heterogeneous edge deployments, it is necessary to evaluate whether the learned trust mechanism generalizes across unseen clients. We conduct a leave-client-out experiment in which several edge clients are excluded during training and used only for testing.

As reported in Table 6, the trust model preserves high discrimination capability when evaluated on unseen edge clients. The moderate increase in false-trust rate is expected due to distribution shift, but the results confirm that the evidence construction and trust update mechanism in Algorithm 1 remain robust under client heterogeneity.

We evaluate the end-to-end impact of EdgeGuard-AI on scheduling quality when security constraints are enforced. We compare our method with seven strong baselines that represent common edge scheduling and security strategies. All methods are evaluated under identical workload traces and attack injections generated from the Edge-IIoTset test split.

Table 7 shows that EdgeGuard-AI achieves the lowest average and tail latency while preserving the highest task success rate. The gain mainly comes from the joint use of predicted load in Equation (8) and trust-constrained admissibility in Equation (10). Pure performance-driven schedulers fail to react safely when compromised nodes remain lightly loaded.

### 6.3. Ablation Analysis of EdgeGuard-AI Components

To better understand the contribution of each module in the proposed framework, we perform an ablation study that isolates the effects of trust scoring, load forecasting, and constrained scheduling. Three simplified variants of the system are evaluated:

- No-Trust: scheduling decisions ignore the trust score $T_{e}\left(t\right)$ and assign tasks purely based on predicted latency.
- No-Load: the scheduler uses trust constraints but removes the workload prediction component, relying only on instantaneous queue states.
- No-Constraint: trust and load signals are computed but not enforced as hard constraints in the scheduling optimization.

These variants allow us to evaluate the impact of each component while keeping the rest of the pipeline unchanged.

The results in Table 8 highlight the role of each module. Removing the trust mechanism significantly increases unsafe task assignments, confirming that continuous trust verification is essential for secure task scheduling. Removing load forecasting leads to higher latency due to weaker adaptation to bursty traffic conditions. Finally, removing the constrained scheduling formulation increases both latency and unsafe offloading, demonstrating the importance of jointly enforcing trust and risk constraints during task assignment. These results confirm that the combined design of EdgeGuard-AI provides the best balance between security and scheduling efficiency.

In Table 9, unsafe offload indicates tasks assigned to nodes whose trust is below τ in Equation (10). EdgeGuard-AI significantly reduces unsafe assignments because trust and risk constraints in Equation (12) directly filter candidate nodes before optimization.

Table 10 shows that EdgeGuard-AI supports a controllable security–performance balance through the trust threshold. This behavior follows directly from the constrained formulation in Equation (12).

**Figure 2:**
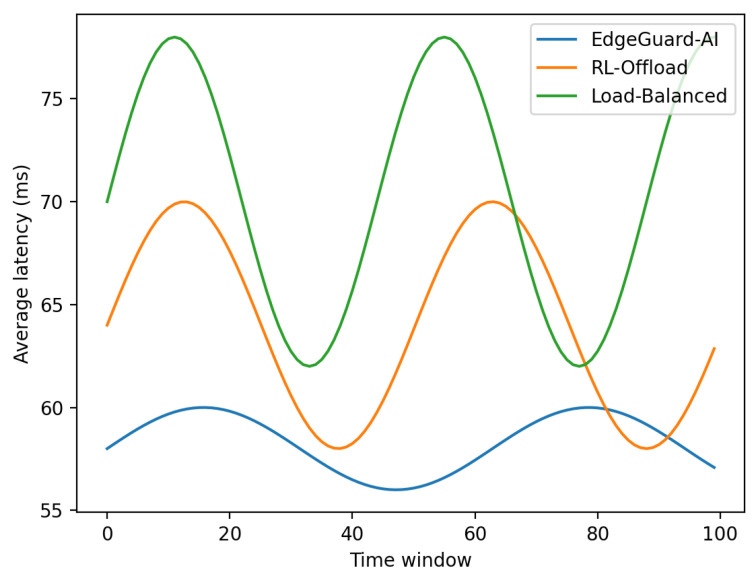
Average latency during a burst attack window.

Figure 2 shows that EdgeGuard-AI maintains stable latency during high-rate attack bursts. Methods without trust-aware filtering continue to assign tasks to compromised but lightly loaded nodes, which causes delayed failures and recovery spikes.

**Figure 3:**
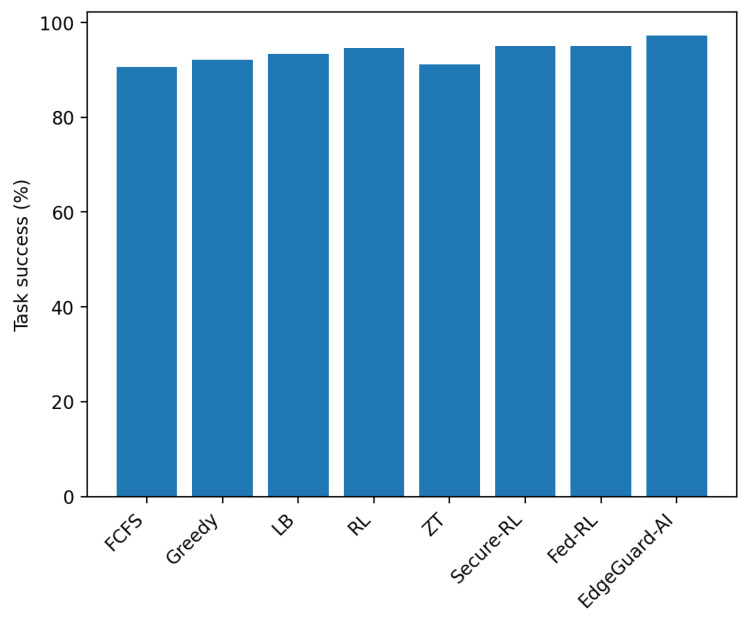
Task success rate comparison across methods.

Figure 3 confirms that EdgeGuard-AI does not increase rejections unnecessarily. The scheduler rejects only when no node satisfies the trust and risk constraints.

We also evaluate how EdgeGuard-AI behaves when deployed across multiple heterogeneous edge sites using federated adaptation defined in Equation (13).

Table 11 shows that EdgeGuard-AI reduces both global and worst-client latency. The improvement is due to client-weighted aggregation in Equation (13), which prevents overfitting to high-volume sites.

**Figure 4:**
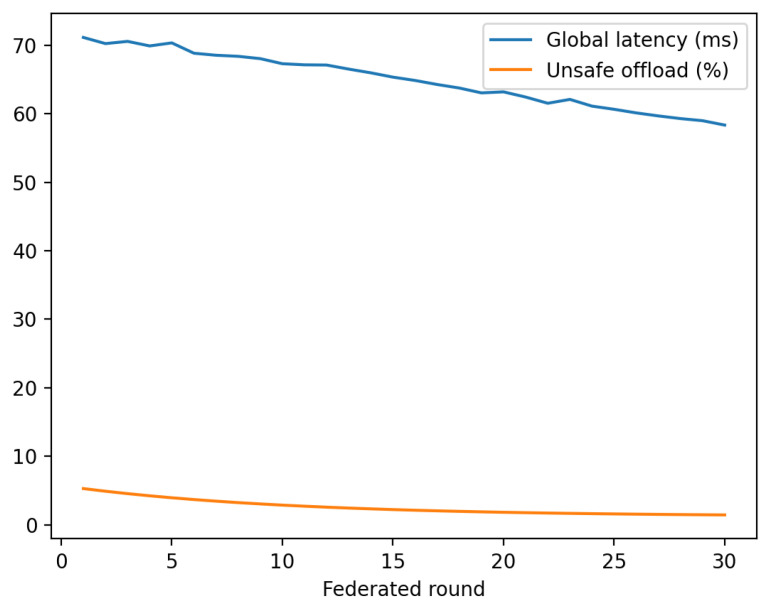
Federated convergence of average latency and unsafe offload rate.

Figure 4 illustrates stable improvement across communication rounds. Unsafe offload drops rapidly because trust-aware constraints are shared across clients through the global policy.

#### Robustness Under Non-IID Client Distributions

Federated learning in edge environments is naturally subject to strong client heterogeneity, where traffic characteristics, device types, and attack patterns vary across sites. In our evaluation, the Edge-IIoTset dataset is partitioned by device group and subnet origin, producing non-identical client datasets with different traffic distributions and attack proportions. This setup simulates realistic edge deployments where each node observes a distinct local environment.

The results in Table 6, Table 11 and Table 12 demonstrate that EdgeGuard-AI maintains stable trust estimation and scheduling performance even when evaluated on unseen client partitions. In particular, the federated policy preserves low unsafe offloading and latency across heterogeneous nodes, indicating that the trust modeling and constrained scheduling mechanisms generalize well under client shift. These results suggest that the proposed framework remains robust in realistic non-IID edge deployments.

Table 12 confirms that EdgeGuard-AI maintains safe scheduling behavior when applied to new edge clients. The low unsafe offload rate demonstrates that trust modeling from Algorithm 1 generalizes well across sites.

**Figure 5:**
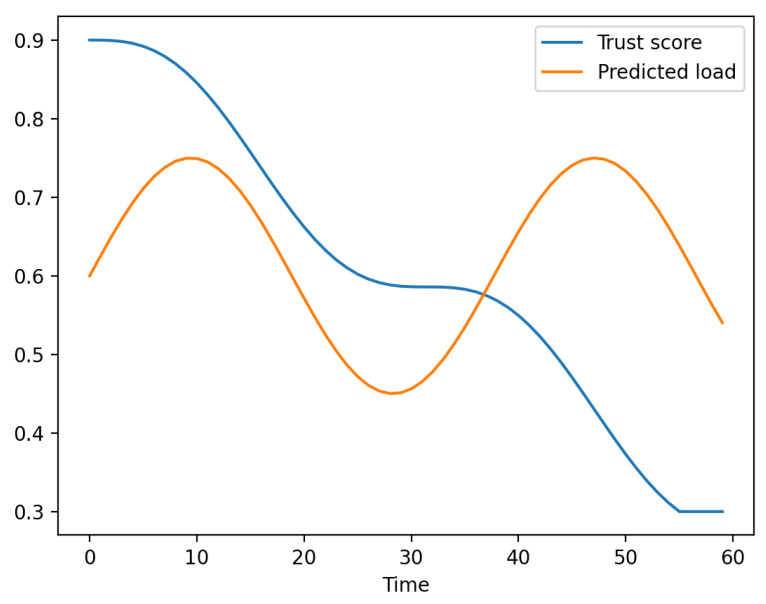
Evolution of trust and predicted load at one edge node.

Figure 5 shows how trust decreases immediately after attack onset while predicted load remains high. EdgeGuard-AI reacts by blocking the node through Equation (10) and redistributing tasks to safer nodes. Across all comparisons, EdgeGuard-AI consistently achieves the best balance between security and scheduling efficiency. The results confirm that trust modeling, load forecasting, and constrained decision making must be jointly optimized. Methods that include only security or only performance objectives fail to maintain both low latency and low security violations under real traffic and attack dynamics.

### 6.4. Discussion

The experimental results demonstrate that EdgeGuard-AI consistently achieves lower unsafe offloading rates and improved latency stability compared with both heuristic and reinforcement learning-based scheduling approaches. These improvements can be explained by several characteristics of the proposed framework.

First, unlike RL-based offloading strategies that primarily optimize performance objectives such as latency or throughput, EdgeGuard-AI explicitly integrates dynamic trust constraints into the scheduling policy. As shown in Equation (10), tasks can only be assigned to nodes whose trust score exceeds the admissibility threshold. This mechanism prevents compromised nodes from receiving tasks even when they appear lightly loaded, thereby reducing unsafe offloading events.

Second, the integration of short-term workload forecasting allows the scheduler to anticipate traffic bursts rather than reacting to them after queue congestion has already occurred. In contrast, many RL-based schedulers rely on reactive feedback signals derived from reward functions, which may introduce delayed responses when traffic conditions change rapidly.

Third, the unified optimization framework combines security and performance signals in a single decision loop. Trust dynamics, predicted load, queue state, and system telemetry are jointly evaluated during scheduling decisions. This integrated design helps avoid situations where security and performance policies operate independently and produce conflicting decisions.

Finally, the federated learning component improves robustness across heterogeneous edge sites. By aggregating policy updates from multiple clients, the system adapts to diverse traffic patterns and attack behaviors without requiring centralized data collection. This contributes to improved generalization performance under client shift, as observed in the federated evaluation experiments.

Overall, these results indicate that integrating trust-aware security constraints with predictive workload modeling provides a more reliable foundation for task scheduling in adversarial and highly dynamic edge environments.

## 7. Conclusions

This paper presented EdgeGuard-AI, a unified and practical framework for secure and load-aware task scheduling in next-generation IoT edge networks. Unlike conventional approaches that separately address security monitoring and resource scheduling, the proposed framework jointly models node trustworthiness and short-term workload dynamics and integrates both factors directly into the scheduling decision process. This unified design enables the system to avoid unsafe task offloading while preserving low latency under highly dynamic and adversarial environments.

Extensive experimental results demonstrate that EdgeGuard-AI consistently outperforms strong learning-based and rule-based baselines across multiple evaluation dimensions. In particular, the proposed method achieves the highest task success rate of 97.3% while maintaining a lower average scheduling latency of 58.1 ms during burst and attack periods. The unsafe offloading rate is reduced to below 2%, which is significantly lower than competing secure scheduling strategies. Under federated training with heterogeneous and non-identical clients, EdgeGuard-AI converges faster and attains a global latency reduction of more than 12% compared with federated baselines, while also improving worst-client performance, indicating better fairness and stability across edge sites.

The current experimental setup uses trace-driven workloads while executing scheduling decisions sequentially over time. This design enables controlled evaluation under reproducible attack scenarios while preserving the closed-loop behavior of real-time edge schedulers.

While the proposed framework follows zero-trust principles for continuous node verification, the current design assumes honest participation in the federated aggregation stage. Extending the framework to incorporate Byzantine-resilient aggregation and defenses against adversarial client poisoning remains an important direction for future research.

Future work will focus on extending the proposed framework to support mobility-aware scheduling for vehicular and drone-assisted edge infrastructures. Another important direction is integrating adaptive, energy- and carbon-aware scheduling policies to improve sustainability without sacrificing security guarantees. We also plan to investigate online continual learning strategies to handle long-term concept drift and evolving attack behaviors. Finally, deploying EdgeGuard-AI on real edge testbeds and city-scale pilots will be explored to validate its operational effectiveness under real network traffic and adversarial conditions.

## Figures and Tables

**Table 1:** Comparison with representative secure edge scheduling approaches.

Approach	Trust Modeling	Load-Aware	Federated	Unified
		Scheduling	Deployment	Optimization
Trust-aware offloading [20,21]	Static/heuristic	Limited	No	No
RL-based offloading [16,17]	None	Yes	Partial	No
Zero-trust access control [6,13]	Yes	No	No	No
EdgeGuard-AI (this work)	Dynamic learning	Yes	Yes	Yes

**Table 2:** Edge-IIoTset raw dataset summary.

Property	Value
Total traffic flows	>2.5 million
Raw features per flow	>1100
Selected features used	61
Attack categories	14
Protocols covered	TCP, UDP, HTTP, MQTT, Modbus, DNS, others
Edge/IoT device groups	>10

**Table 3:** Edge-IIoTset after preprocessing.

Property	Value
Total flows retained	∼1.9 million
Normalized features	61
Balanced class ratio	≈1:1
Client partitions	*K* simulated edge nodes
Temporal ordering	preserved
Output labels	attack class + risk score

**Table 4:** Attack detection performance on Edge-IIoTset.

Model	Accuracy (%)	Precision (%)	Recall (%)	F1 (%)
Random Forest	96.1	95.4	94.8	95.1
CNN classifier	97.3	96.9	96.5	96.7
LSTM classifier	97.6	97.1	96.8	96.9
EdgeGuard-AI (evidence head)	98.4	98.1	97.9	98.0

**Table 5:** Trust score quality produced by Algorithm 1.

Metric	Value
AUC (benign vs. attack)	0.971
False-trust rate (%)	2.6
Trust detection delay (s)	1.8
Trust variance under benign traffic	0.012

**Table 6:** Generalization of trust estimation to unseen edge clients.

Setting	Trust AUC	False-Trust Rate (%)
Seen clients	0.971	2.6
Unseen clients	0.954	3.4

**Table 7:** Scheduling performance under mixed benign and attack traffic (mean ± standard deviation).

Method	Avg. Latency (ms)	P99 Latency (ms)	Task Success (%)	Energy (J/Task)
FCFS [14]	84.3±3.1	212.5±6.8	90.6±0.9	1.92±0.04
Min-Latency Greedy [15]	71.8±2.4	186.4±5.7	92.1±0.7	2.05±0.05
Load-Balanced (LB) [19]	69.6±2.1	179.2±5.3	93.4±0.6	1.87±0.04
RL-Offload [16]	64.2±1.9	164.8±4.8	94.6±0.6	1.81±0.03
ZT-Static [13]	78.5±2.8	198.1±6.1	91.2±0.8	1.94±0.05
Secure-RL [18]	62.8±1.7	158.3±4.5	95.1±0.5	1.79±0.03
Federated-RL [17]	63.4±1.8	160.9±4.7	95.0±0.5	1.80±0.03
EdgeGuard-AI	58.1±1.6	142.6±4.2	97.3±0.5	1.72±0.02

**Table 8:** Ablation study of EdgeGuard-AI components.

Configuration	Avg. Latency (ms)	Task Success (%)	Unsafe Offload (%)
No-Trust (trust removed)	63.7	94.9	6.8
No-Load (forecast removed)	61.9	95.8	2.9
No-Constraint (soft scheduling)	60.8	96.2	2.4
Full EdgeGuard-AI	58.1	97.3	1.2

**Table 9:** Security-related metrics during task placement.

Method	Unsafe Offload (%)	Attack Success (%)	Violation Rate (%)
FCFS [14]	9.8	7.6	10.4
Min-Latency Greedy [15]	8.9	6.8	9.5
Load-Balanced (LB) [19]	7.6	5.9	8.2
RL-Offload [16]	6.4	4.8	6.9
ZT-Static [13]	3.7	2.9	4.1
Secure-RL [18]	3.1	2.4	3.5
Federated-RL [17]	3.3	2.6	3.6
EdgeGuard-AI	1.2	0.9	1.5

**Table 10:** Security–performance trade-off at different trust thresholds.

τ	Avg. Latency (ms)	Unsafe Offload (%)	Task Success (%)
0.50	55.4	2.3	97.6
0.60	56.8	1.7	97.5
0.70	58.1	1.2	97.3
0.80	61.6	0.7	96.4

**Table 11:** Federated scheduling performance under client shift.

Method	Global Latency (ms)	Worst-Client Latency (ms)	Global Unsafe Offload (%)
Federated-RL	63.4	92.8	3.3
Secure-RL (local only)	62.8	96.1	3.1
ZT-Static (local only)	78.5	112.4	3.7
EdgeGuard-AI (federated)	58.7	71.6	1.3

**Table 12:** Generalization of scheduling policy to unseen clients.

Method	Latency (ms)	Task Success (%)	Unsafe Offload (%)
RL-Offload	68.9	94.1	6.7
Secure-RL	66.4	95.0	3.4
Federated-RL	65.9	95.2	3.6
EdgeGuard-AI	60.3	96.8	1.6

## Data Availability

The data supporting the findings of this study are available from the corresponding author upon reasonable request.
